# Long‐term survival outcomes following laparoscopic surgery for clinical stage 0/I rectal carcinoma

**DOI:** 10.1002/ags3.12333

**Published:** 2020-05-20

**Authors:** Masaaki Ito, Seiichiro Yamamoto, Junji Okuda, Shoichi Fujii, Shigeki Yamaguchi, Koki Otsuka, Kenichi Yoshimura, Masahiko Watanabe, for the Japan Society of Laparoscopic Colorectal Surgery

**Affiliations:** ^1^ Department of Colorectal Surgery National Cancer Center Hospital East Kashiwa Japan; ^2^ Department of Surgery Tokai University School of Medicine Hiratsuka Japan; ^3^ Department of Generl and Gastroenterological Surgery Osaka Medical College Takatsuki Japan; ^4^ Department of Surgery Sunkokai Koga Community Hospital Yaizu Japan; ^5^ Department of Gastroenterological Surgery Saitama Medical University International Medical Center Hidaka Japan; ^6^ Department of Surgery Iwate Medical University School of Medicine Morioka Japan; ^7^ Center for Integrated Medical Research Hiroshima University Hospital Higashihiroshima Japan; ^8^ Department of Surgery Kitasato Institute Hospital Kitasato University Tokyo Japan

**Keywords:** clinical stage I, clinical trial, laparoscopy, long‐term outcome, rectal cancer

## Abstract

**Aim:**

To clarify and evaluate the long‐term outcomes of laparoscopic surgery for clinical stage 0/I rectal carcinoma patients.

**Methods:**

This single‐arm phase II trial involved accredited surgeons from 43 Japanese institutions. Patients were registered preoperatively. The planned sample size was 490. The primary endpoint was overall survival, and long‐term outcomes were evaluated.

**Results:**

A total of 495 patients were registered between February 2008 and August 2010. Eight patients (1.6%) required conversion to open surgery. Sphincter‐preserving procedures were performed in 477 (97%) patients. Positive radial resection margin was found in two (0.4%) patients. Of 490 patients, 22, 314, 38, 115, and one patient had final pathological stages (p‐stage) 0, I, II, III, and IV, respectively. Pathologically, 31.4% (154/490) of the patients did not have p‐stage 0/I. The 5‐year overall survival (OS) rates in p‐stages 0, I, II, and III were 100%, 98%, 97%, and 94%, respectively. The 5‐year OS of all patients at 96.6% (95% CI 94.6‐97.9) was significantly better than the expected 5‐year OS of 81.1% (*P *< .0001). The 5‐year relapse‐free survival in p‐stages 0, I, II, and III were 100%, 93%, 81%, and 79%, respectively. The 5‐year relapse‐free survival of all patients was 90.1%. Fifty patients (10.2%) had recurrence; lung recurrence was found in 22 patients, local recurrence in 14, liver in seven, distant lymph node in nine, and bone in three.

**Conclusions:**

Laparoscopic surgery for clinical stage 0/I rectal carcinoma has feasible long‐term outcomes. (ClinicalTrials.gov No.NCT00635466.)

## Graphical abstract

This single‐arm phase II trial involved accredited surgeons from 43 Japanese institutions. The primary endpoint was overall survival, and long‐term outcomes were evaluated. Laparoscopic surgery for clinical stage 0/I rectal carcinoma has feasible long‐term outcomes.

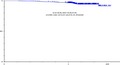

## 1 INTRODUCTION

Total mesorectal excision (TME), introduced by Heald and Ryall^1^ in the 1980s, has remained the gold standard surgical treatment for rectal cancer. Preoperative chemoradiotherapy (CRT) following TME has improved clinical results of locoregional recurrence rates to below 10%,^2^, ^3^, ^4^, ^5^ and these combinations of TME and CRT are still widely used as the main axis of rectal cancer surgical treatment.

Laparoscopic surgery began to be introduced clinically from the 1990s, and its equivalence with laparotomy surgery has been verified by some randomized control trials.^6^, ^7^, ^8^ As a result, laparoscopic surgery for colon cancer had equal long‐term survival rates to open surgery as well as favorable short‐term outcomes. Laparoscopic surgery for rectal cancer required a narrow pelvic operation and complex procedures compared to colon cancer had been indicated to have some potential risks for curative resection.^7^ The COLOR II trial and COREAN trial successfully showed that laparoscopic surgery in rectal cancer patients had similar locoregional recurrence, disease‐free survival (DFS), and OS rates to open surgery.^9^, ^10^ However, two recent randomized trials showed that positive circumferential resection rate (CRM) in the laparoscopic surgery group was higher than that in the open surgery group for rectal cancer, and whether laparoscopic surgery for rectal cancer has feasible long‐term survival remains to be discussed. Laparoscopic rectal excision has technically demanding aspects, which might be the reasons for the higher anastomotic leakage rate or worse curative resection rate in case of stage II/III rectal cancer with estimated CRM < 1 mm.^11^

In the 2000s, the usefulness of laparoscopic surgery for rectal carcinoma remains uncertain because of concerns over the safety of the procedure, especially low anterior resection for middle or lower rectal carcinoma. Therefore, we conducted a clinical trial in patients with a preoperative diagnosis of relatively early‐stage rectal carcinoma to examine the technical and oncological feasibility of laparoscopic surgery for rectal carcinoma. Our past report showed feasible short‐term results including an anastomotic leakage rate of 8%.^12^

As a final report, the aim of this study was to clarify the long‐term oncological results after 5 years, conducted under the direction of the Japan Society of Laparoscopic Colorectal Surgery, of which the leading hospitals in laparoscopic surgery for colorectal carcinoma in Japan are members.

## 2 METHODS

### 2.1 Study design and participants

The inclusion criteria have been reported previously.^13^ Eligibility criteria included histologically proven rectal cancer, clinically diagnosed as Tis‐T2/N0/M0 lesions on the basis of colonoscopy, pelvic computed tomography, transanal ultrasonography, or magnetic resonance imaging. When the tumor was located between the inferior margin of the second sacral vertebra and the peritoneal reflection, the location was recorded as the upper rectum. When the tumor was located below the peritoneal reflection, its location was recorded as the lower rectum.^14^ Tumor location was determined by pelvic computed tomography, colonoscopy, and/or barium enema preoperatively and was confirmed during surgery.

The study protocol was approved by the Ethics Committee of the Japanese Society for Cancer of the Colon and Rectum and approved and overseen by the institutional review board of each participating hospital. All patients gave written informed consent.

This confirmatory, multi‐institutional, nonrandomized, single‐arm (laparoscopic) trial (phase II) was conducted to evaluate short‐ and long‐term outcomes of laparoscopic surgery for clinical stage (c‐stage) 0/I rectal carcinoma. Patients were recruited at 43 specialized centers in Japan. The primary endpoint in the study is OS, and patients are still being followed for this endpoint.

### 2.2 Registration

Eligible patients were registered preoperatively by calling the registration office at Kitasato University after confirmation of the inclusion/exclusion criteria. An eligibility report form was sent to the Data Center at the Clinical Trial Coordinating Office at the National Cancer Center Hospital.

### 2.3 Procedures and quality control

Surgery was performed by 61 accredited surgeons. Surgeons with experience of more than 30 laparoscopic and 30 open operations for rectal carcinoma were accredited by the study chair to participate in this study. We performed a central review of the surgical procedure by photograph in all patients and by video in arbitrarily selected patients.

Surgical procedures were performed as described previously.^13^ In brief, laparoscopic resection of the rectum with adequate lymphadenectomy was performed. The extent of lymphadenectomy and ligation site and division of the inferior mesenteric vessels were decided by the surgeon in charge.

Mobilization of the rectum, excision of the mesorectum, rectal transection, removal of the specimen, and reconstruction were performed by pneumoperitoneal approach or extracorporeal approach via an incision smaller than 8 cm. Bowel anastomoses were performed intracorporeally via a small incision using the double stapling technique or by transanal hand‐sewn sutures.

For sphincter‐preserving operations, the decision to make a protective ileostomy was based on the surgeon's technical evaluation of the quality of anastomosis. When an incision longer than 8 cm was required for the control of intraoperative complications or tumor extension, the operation was considered a conversion. Operative methods and pathology results were recorded according to the Japanese Classification of Colon and Rectal Carcinoma (sixth edition) and TNM classification (sixth edition).^14^, ^15^ For surveillance after curative surgery, blood tests, abdominal and pelvic computed tomographic scans, and plain chest radiographs were obtained at each visit, and colonoscopy at 1, 3, and 5 years after the operation was carried out.

### 2.4 Statistical analysis

In this study, the expected 5‐year OS rate was 88% and the threshold value was 83%. The sample size was originally estimated as 350 (one‐sided α = 0.05 and β = 0.2) with an expected pathological stage (p‐stage) I:II:III ratio of 0.8:0.1:0.1; however, we examined the p‐stage I:II:III ratio at the analysis of the first stage, and it was 0.70:0.08:0.22. As expected, the number of p‐stage I patients was limited; hence, the sample size was increased to 490 patients to maintain the required statistical power. The planned accrual period was 3 years, and the follow‐up period was set at 5 years after completion of accrual. This study is registered with ClinicalTrials.gov No. NCT00635466.

## 3 RESULTS

### 3.1 Patients and short‐term outcomes

A total of 495 patients were registered between February 2008 and August 2010. Five patients were ineligible after registration. After their exclusion, 490 patients were included in the long‐term analysis.

The number of patients with c‐stage Tis, T1, and T2 was two, 291, and 197, respectively. Overall, 336 patients (68.6%) had final p‐stage 0/I and 38, 115, and one patient had p‐stages II, III, and IV, respectively. The median number of harvested lymph node was 17. Positive resection margin which was defined as exposed cancer cells in the resected margin was found in two patients (0.4%; Table 1).

**TABLE 1:** Clinical and pathological characteristics of the patients

Characteristic	
Total number of patients	490
Sex ‐ no (%)	
Male	281 (57.3)
Female	209 (42.7)
Age ‐ yr; mean ± SD	59.7 ± 9.8
American Society of Anesthesiologists classification ‐ no (%)	
I	356 (72.7)
II	134 (27.3)
Body mass index ‐ kg/m^2^, mean ± SD	22.7 ± 3.2
Tumor location ‐ no (%)	
Upper rectum	218 (44.5)
Lower rectum	272 (55.5)
Clinical T stage ‐ no (%)	
Tis	2 (0.4)
T1	291 (59.4)
T2	197 (40.2)
Pathological stage ‐ no (%)	
Stage 0	22 (4.5)
Stage I	314 (64.1)
Stage II	38 (7.8)
Stage III	115 (23.4)
Stage IV	1 (0.2)
Lymph nodes harvested	17
Resection margin ‐ no (%)	
Positive	2 (0.4)
Negative	488 (99.6)

The median operative time was 270 minutes, and median blood loss was 28 mL. Operations in eight (1.6%) patients were converted to open surgery. Sphincter‐preserving surgeries were possible in 477 (97%) patients, including 77 (15.7%) patients. Intersphincteric resection (ISR) was also performed (Table 2).

**TABLE 2:** Operative results

Laparoscopic procedures ‐ no (%)	
Anterior resection	400 (81.6)
Intersphincteric resection	77 (15.7)
Abdominoperineal resection	12 (2.4)
Abdominosacral resection	1 (0.2)
Temporaｒy stoma at the first operation ‐ no (%)	
Anterior resection	89 (18.1)
Intersphincteric resection	68 (13.8)
Operative time, median (range), min	270 (110‐565)
Blood loss, median (range), mL	28 (1‐2103)
Conversion ‐ no (%)	8 (1.6)

### 3.2 OS and DFS

**FIGURE 1:**
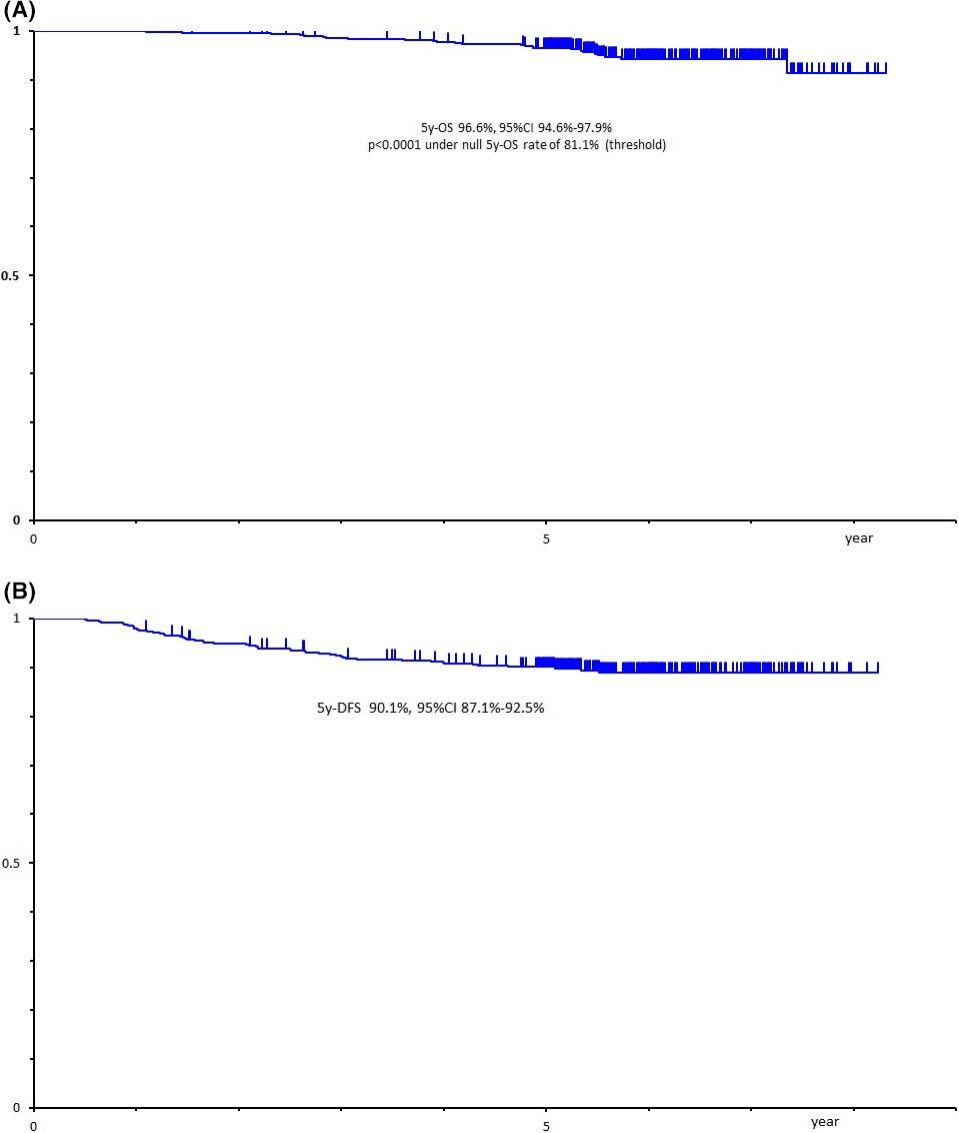
(A) Overall survival. (B) Disease‐free survival

**FIGURE 2:**
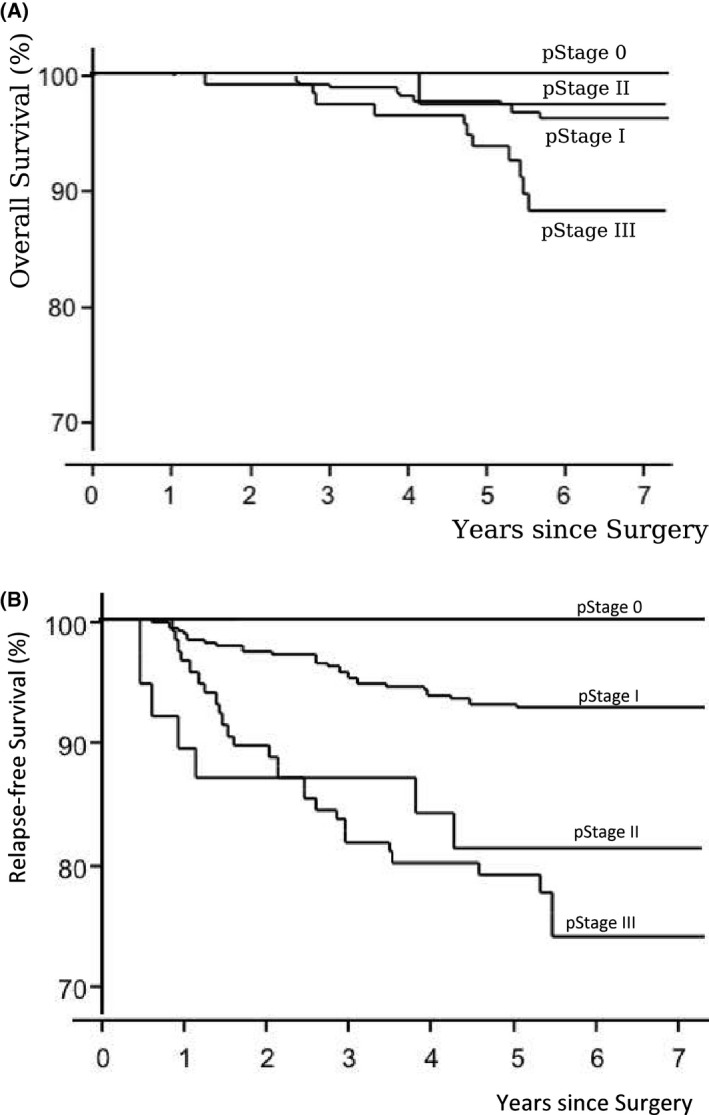
(A) Overall survival of laparoscopic surgery by pathological stages. (B) Relapse‐free survival of laparoscopic surgery by pathological stages

The 5‐year OS rate of all patients was 96.6% (95% CI 94.6‐97.9), which was significantly better than the expected 5‐year OS of 81.1% in the initial plan of the protocol (*P *< .0001) (Figure 1A). The 5‐year relapse‐free survival (RFS) rate of all patients was 90.1% (Figure 1B). The 5‐year OS rates in p‐stages 0, I, II, and III were 100%, 98%, 97%, and 94%, respectively (Figure 2A), and the 5‐year RFS rates in p‐stages 0, I, II, and III were 100%, 93%, 81%, and 79%, respectively (Figure 2B). The total number of patients with recurrence was 50 (10.2%). Lung recurrence was found in 22 patients, local recurrence in 14, liver in seven, distant lymph node in nine, and bone in three.

### 3.3 Locoregional recurrence

Among all 490 cases, locoregional recurrence was found in 14 cases (2.8%). Three cases of local recurrences were found in mesorectal lymph node, three in lateral lymph node, two in anastomotic site, two in presacral region, and four were unknown.

In the p‐stage, locoregional recurrence was observed in five cases in p‐stage I, one case in stage II, and eight cases in stage III. Analysis of each rectal site revealed locoregional recurrence in three cases (1.4%) located in the upper rectum. Locoregional recurrence was observed in 11 cases in the lower rectal cancer, which included four cases (2.1%) in stage 1, one case (7.7%) in stage 2, and six cases (10.7%) in stage 3. According to the operation procedure, locoregional recurrence was confirmed in eight of 400 cases (2.0%) of anterior resection and in six of 77 cases (7.8%) of ISR (Table 3).

**TABLE 3:** Locoregional recurrence

Tumor location and procedure (n)	Number of locoregional recurrence (%)
Tumor location^a^	
Total rectum	
All stage (490)	14 (2.8%)
p‐stage 0 (22)	0 (0%)
p‐stage I (314)	5 (1.6%)
p‐stage II (38)	1 (2.6%)
p‐stage III (115)	8 (7.0%)
Upper rectum	
All stage (218)	3 (1.4%)
p‐stage 0 (12)	0 (0%)
p‐stage I (122)	1 (0.8%)
p‐stage II (25)	0 (0%)
Low rectum	
All stage (272)	11 (4.0%)
p‐stage 0 (10)	0 (0%)
p‐stage I (192)	4 (2.1%)
p‐stage II (13)	1 (7.7%)
p‐stage III (56)	6 (10.7%)
Operative procedure	
Anterior resection (400)	8 (2.0%)
Intersphincteric resection (77)	6 (7.8%)
Abnomino‐perineal resection (12)	0 (0%)

^a^ Data of p‐stage IV was excluded in this table due to only one case.

## 4 DISCUSSION

### 4.1 Summary

In the 2000s, non‐inferiority of the long‐term oncological outcome between open surgery and laparoscopic surgery was demonstrated by several randomized trials for colon cancer. However, laparoscopic surgery for rectal cancer is still a challenging procedure, and our Japanese study group conducted a clinical, one‐arm, phase II trial to clarify the short‐ and long‐term results of laparoscopic surgery for c‐stage 0/I low rectal cancer. The primary endpoint of this study was 5‐year OS rate of 96.6%, which was higher than the estimated 5‐year OS rate of 81.1%, and this long‐term survival rate could meet the primary endpoint in the current study.

### 4.2 Comparison of eligibility with randomized control trials

At present, results of four large randomized control trials on laparoscopic rectal cancer surgery have been published.^9^, ^10^, ^16^, ^17^ In particular, two recent randomized trials from Australia and the USA demonstrated higher positive CRM rates in the laparoscopic group than the open group.^16^, ^17^ Therefore, the safety of laparoscopic surgery for rectal cancer still needs to be established, and the potential risk of impaired surgical quality should be recognized. Four large randomized trials, namely, COLOR II trial, COREAN trial, ALaCaRT trial, and ACOSOG Z6051, included rectal cancer patients with T1 to T3 and N0 to N2, located within 12‐15 cm from the anal verge, and preoperative CRT was introduced in 50%‐100% of the patients in these studies as standard therapy.

Strictly, eligible patients in the COREAN trial and ACOSOG trial were limited to those with c‐stages II and III, but 201 patients with c‐stage I were included in the COLOR II trial and some patients in the ALaCaRT trial. Moreover, the patient backgrounds of these four randomized controlled trials varied. Similarly, the inclusion criteria in tumor location are different among the studies. The eligibility criteria of the COLOR II trial and ALaCaRT trial included tumors within 15 cm from the anal margin, which ranged from the upper to the lower rectum. On the contrary, the COREAN trial is limited to the mid‐low rectum. According to the current Japanese classification of colorectal, appendiceal, and anal carcinoma, lower rectal cancer is defined as having a tumor below the peritoneal reflex and the upper rectum is defined as the location between the peritoneal reflex and the lower border of the second sacrum. That is, according to the present Japanese definition, the Japanese upper rectum is obviously different from the upper rectum defined in other international trials and may be interpreted as being close to the middle rectum. Inclusion criteria in this study correspond to the lower two‐thirds of the rectum, which was globally recognized as middle or lower rectum. Therefore, our clinical trial can be interpreted as not significantly different from other tests in terms of tumor location.

### 4.3 Interpretation of local recurrence

CRM has been used as an index for evaluating the curability of rectal cancer surgery. A study reported that CRM predicted by preoperative MRI can distinguish curability,^18^ requires at least 1 mm CRM after preoperative radiochemotherapy,^19^ and more than 2 mm CRM is necessary for surgery alone.^20^ A positive CRM could predispose patients to locoregional recurrence, as observed in 10% of the patients after laparoscopic surgery in the COLOR II trial, in 3% in the COREAN trial, and in 7% in the ALaCaRT trial. Given the limited number of institutions that measure CRM from pathological specimens in Japan at the time of the protocol preparation, we could not evaluate CRMs. Instead, two cases (0/4%) of all cases in this study where tumor cells were clearly exposed by surgical margin were observed in our pathological evaluation. No locoregional recurrences were observed in two cases with positive surgical margin.

The 5‐year locoregional recurrence rate in our study was 2.8%, although all c‐stage 0/I rectal cancer cases were treated by surgery alone. On the contrary, the 3‐year locoregional recurrence rates in the COLOR II trial and COREAN trial were 5.0% and 2.6%, respectively. In our study, the 5‐year locoregional recurrence rate was 1.4% for upper rectal cancer and 4.0% for lower rectal cancer after laparoscopic surgery. In the subgroup analysis of this study, relatively high 5‐year locoregional recurrence rates were observed in p‐stage II lower rectal cancer (7.7%), p‐stage III lower rectal cancer (10.7%), and ISR patients (7.8%), who might require preoperative CRT before surgery.

### 4.4 Comparison of DFS

In this study, 153 patients with p‐stage II/III were included. The 5‐year RFS in patients diagnosed with p‐stage II and III were 81% and 79% and the 5‐year OS in p‐stage II and III were 97% and 94%, respectively. The 5‐year survival rate for patients with more than p‐stage II seemed to be good in this study. However, it may be due to the bias that all included patients were diagnosed as clinical stage I.

The 3‐year DFS in the laparoscopic group with p‐stage II/III was evaluable in both COLOR II trial and COREAN trial, which showed 74.8% and 79.2%, respectively. Moreover, the 3‐year OS rate was 86.7% in the COLOR II trial and 91.7% in the COREAN trial. In the COLOR II trial, analyzed only for p‐stage III, the 3‐year DFS of laparoscopic surgery was 64.9%. In our study, the 3‐year DFS of laparoscopic surgery for p‐stage III was 79%. Since the patients in our study were preoperatively diagnosed with c‐stage 0/1 and underwent laparoscopic surgery without preoperative CRT, long‐term outcomes in p‐stage II/III were equal or had better survival rates compared with other international randomized trials.

### 4.5 Comparison of long‐term survival rates with other Japanese trials

A recent Japanese randomized control trial (JCOG0212) comparing mesorectal excision (ME) and ME plus lateral lymph node dissection showed 5‐year OS and 5‐year RFS rates in c‐stage II/III lower rectal cancer treated by open surgery.^21^, ^22^ The 5‐year OS and 5‐year RFS rates in c‐stage II/III low rectal cancer in JCOG0212 were 90.2% and 73.3%, respectively, treated by ME alone without preoperative treatment. The current study included 152 patients with p‐stage II/III treated by surgery alone, which indicated a 5‐year RFS rate of 81% in p‐stage II and 79% in p‐stage III and 5‐year OS rate of 97% in p‐stage II and 94% in p‐stage III. The long‐term survival rate of laparoscopic surgery for rectal cancer with p‐stages II and III in this study, despite the direct and careful comparison of the results of two different clinical trials, was not worse than that of conventional surgery for rectal cancer shown in JCOG0212 study.

### 4.6 Limitation of this study

A limitation of our study is the absence of randomized study design between laparoscopic surgery and open surgery, as our study group included 43 specialized hospitals for colorectal surgery, in which laparoscopic experts had already performed laparoscopic surgery for early‐staged rectal cancer as clinical practice. Therefore, since we planned the study design, it was difficult to conduct a randomized trial comparing open surgery and laparoscopic surgery.

This is the first control study that focused on rectal cancer with c‐stage I, and we could have accumulated a large number of cases, although the study only included laparoscopic surgery group as a one‐arm study. Long‐term results in laparoscopic surgery are appropriate for rectal cancer preoperatively diagnosed as at least c‐stage I. However, our study also indicated that locoregional recurrence rates in lower rectal cancer with p‐stage II/III and ISR patients were around 7%‐10%. Especially, in ISR, CRM shortage is a concern because of the required laparoscopic skill and understanding of the pelvic anatomy. In these cases, it may be necessary to consider other therapeutic options for preoperative CRT and conversion to abdominoperineal resection. Currently, we are conducting a phase II study in Japan for patients with rectal cancer closed to the anal canal, and we are waiting for the results on locoregional recurrence rate and function preservation.

## 5 CONCLUSIONS

Recent results indicate that stage II/III low rectal cancers are associated with some factors that limit the performance of safe surgery with curative content. C‐stage I low rectal cancer is a reasonable indication, and we could, therefore, offer safe operation for low rectal cancer. In conclusion, laparoscopic surgery for c‐stage 0/I rectal carcinoma has feasible long‐term outcomes.

## DISCLOSURE

Conflict of Interest: The authors declare no conflicts of interest.

## References

[1] Heald RJ , Ryall RD . Recurrence and survival after total mesorectal excision for rectal cancer. Lancet. 1986;1:1479–82. 2425199 10.1016/s0140-6736(86)91510-2

[2] MacFarlane JK , Ryall RD , Heald RJ . Mesorectal excision for rectal cancer. Lancet. 1993;341:457–60. 8094488 10.1016/0140-6736(93)90207-w

[3] Kapiteijn E , Marijnen CA , Nagtegaal ID , Putter H , Steup WH , Wiggers T , et al. Preoperative radiotherapy combined with total mesorectal excision for resectable rectal cancer. N Engl J Med. 2001;345:638–46. 11547717 10.1056/NEJMoa010580

[4] Laurent C , Leblanc F , Wütrich P , Scheffler M , Rullier E . Laparoscopic versus open surgery for rectal cancer: long‐term oncologic results. Ann Surg. 2009;250:54–61. 19561481 10.1097/SLA.0b013e3181ad6511

[5] Jeong SY , Park JW , Nam BH , Kim S , Kang SB , Lim SB , et al. Open versus laparoscopic surgery for midrectal or low‐rectal cancer after neoadjuvant chemoradiotherapy (COREAN trial): survival outcomes of an open‐label, noninferiority, randomised controlled trial. Lancet Oncol. 2014;15:767–74. 24837215 10.1016/S1470-2045(14)70205-0

[6] Fleshman J , Sargent DJ , Green E , Anvari M , Stryker SJ , Beart RW , et al. Laparoscopic colectomy for cancer is not inferior to open surgery based on 5‐year data from the COST Study Group trial. Ann Surg. 2007;246:655–62. 17893502 10.1097/SLA.0b013e318155a762

[7] Jayne DG , Guillou PJ , Thorpe H , Quirke P , Copeland J , Smith AMH , et al. Randomized trial of laparoscopic‐assisted resection of colorectal carcinoma: 3‐year results of the UK MRC CLASICC Trial Group. J Clin Oncol. 2007;25:3061–8. 17634484 10.1200/JCO.2006.09.7758

[8] Lacy AM , García‐Valdecasas JC , Delgado S . Laparoscopy‐assisted colectomy versus open colectomy for treatment of non‐metastatic colon cancer: a randomised trial. Lancet. 2002;359:2224–9. 12103285 10.1016/S0140-6736(02)09290-5

[9] Bonjer HJ , Deijen CL , Haglind E , COLOR II Study Group . A randomized trial of laparoscopic versus open surgery for rectal cancer. N Engl J Med. 2015;373:194. 10.1056/NEJMc1505367 26154803

[10] Jeong SY , Park JW , Nam BH , Kim S , Kang SB , Lim SB ,, et al. Open versus laparoscopic surgery for mid‐rectal or low‐rectal cancer after neoadjuvant chemoradiotherapy (COREAN trial): survival outcomes of an open‐label, non‐inferiority, randomised controlled trial. Lancet Oncol. 2014;15:767–74. 24837215 10.1016/S1470-2045(14)70205-0

[11] Taylor FG , Quirke P , Heald RJ , Moran BJ , Blomqvist L , Swift IR , et al. Preoperative magnetic resonance imaging assessment of circumferential resection margin predicts disease‐free survival and local recurrence: 5‐year follow‐up results of the MERCURY study. J Clin Oncol. 2014;32(1):34–43. 24276776 10.1200/JCO.2012.45.3258

[12] Yamamoto S , Ito M , Okuda J , Fujii S , Yamaguchi S , Yoshimura K , et al. Laparoscopic surgery for stage 0/I rectal carcinoma: short‐term outcomes of a single‐arm phase II trial. Ann Surg. 2013;258:283–8. 23426337 10.1097/SLA.0b013e318283669c

[13] Yamamoto S , Yoshimura K , Konishi F , Watanabe M . Phase II trial to evaluate laparoscopic surgery for stage 0/I rectal carcinoma. Jpn J Clin Oncol. 2008;38:497–500. 18586667 10.1093/jjco/hyn054

[14] Japanese Society for Cancer of the Colon and Rectum . General rules for clinical and pathological studies on cancer of the colon, rectum and anus. 7th ed Tokyo, Japan: Kanehira‐Syuppan; 2006.

[15] Sobin LH , Wittekind C . TNM classification of malignant tumors. 6th ed New York, NY: Wiley‐Liss; 2002.

[16] Stevenson AR , Solomon MJ , Lumley JW , Hewett P , Clouston AD , Gebski VJ , et al. Effect of laparoscopic‐assisted resection vs open resection on pathological outcomes in rectal cancer: the ALaCaRT Randomized Clinical Trial. JAMA. 2015;314:1356–63. 26441180 10.1001/jama.2015.12009

[17] Fleshman J , Branda M , Sargent DJ , Boller AM , George V , Abbas M , et al. Effect of laparoscopic‐assisted resection vs open resection of stage II or III rectal cancer on pathologic outcomes: the ACOSOG Z6051 Randomized Clinical Trial. JAMA. 2015;314:1346–55. 26441179 10.1001/jama.2015.10529 PMC5140087

[18] Taylor FGM , Quirke P , Heald RJ , Moran B , Blomqvist L , Swift I , et al. Preoperative high‐resolution magnetic resonance imaging can identify good prognosis stage I, II, and III rectal cancer best managed by surgery alone: a prospective, multicenter, European study. Ann Surg. 2011;253(4):711–9. 21475011 10.1097/SLA.0b013e31820b8d52

[19] Trakarnsanga A , Gonen M , Shia J , Goodman KA , Nash GM , Temple LK , et al. What is the significance of the circumferential margin in locally advanced rectal cancer after neoadjuvant chemoradiotherapy? Ann Surg Oncol. 2013;20(4):1179–84. 23328971 10.1245/s10434-012-2722-7 PMC4067458

[20] Nagtegaal ID , Marijnen CA , Kranenbarg EK , van de Velde CJH , van Krieken JHJM . Circumferential margin involvement is still an important predictor of local recurrence in rectal carcinoma: not one millimeter but two millimeters is the limit. Am J Surg Pathol. 2002;26(3):350–7. 11859207 10.1097/00000478-200203000-00009

[21] Fujita S , Akasu T , Mizusawa J , Saito N , Kinugasa Y , Kanemitsu Y , et al. Postoperative morbidity and mortality after mesorectal excision with and without lateral lymph node dissection for clinical stage II or stage III lower rectal cancer (JCOG0212): results from a multicentre, randomised controlled, non‐inferiority trial. Lancet Oncol. 2012;13:616–21. 22591948 10.1016/S1470-2045(12)70158-4

[22] Fujita S , Mizusawa J , Kanemitsu Y , Ito M , Kinugasa Y , Komori K , et al. Mesorectal excision with or without lateral lymph node dissection for clinical stage II/III lower rectal cancer (JCOG0212): a multicenter, randomized controlled, noninferiority trial. Ann Surg. 2017;266:201–7. 28288057 10.1097/SLA.0000000000002212

